# Facile Synthesis of Magnetic Iron-Based Nanoparticles from the Leach Solution of Hyperaccumulator Plant *Pinus brutia* for the Antibacterial Activity and Colorimetric Detection of Ascorbic Acid

**DOI:** 10.1021/acsabm.2c00782

**Published:** 2022-10-25

**Authors:** Deniz Uzunoğlu, Ayla Özer

**Affiliations:** Chemical Engineering Department, Mersin University, Çiftlikköy Campus, 33110Mersin, Turkey

**Keywords:** hyperaccumulator plant (Pinus brutia), iron-based nanoparticles, colorimetric ascorbic acid detection, peroxidase-like catalyst, antibacterial nanoparticles

## Abstract

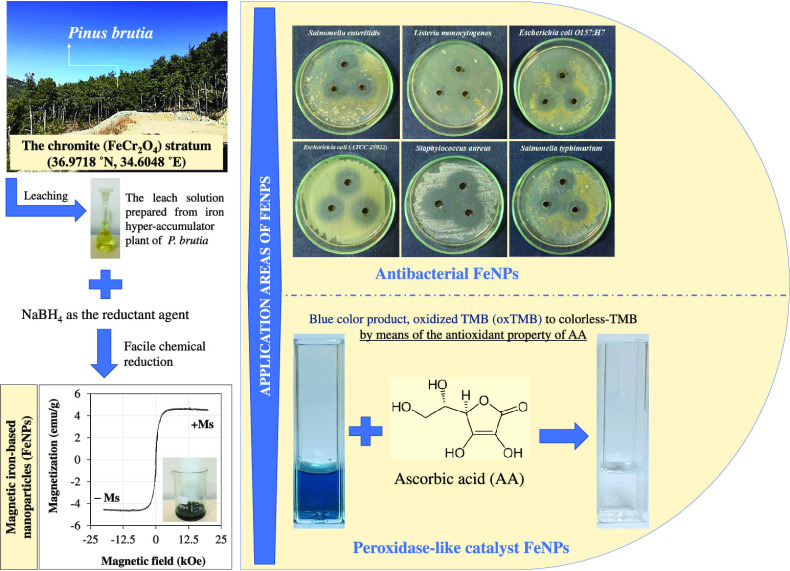

It has been well known that metallic nanoparticles with striking properties possess wide application prospects in the processes of colorimetric detection, catalysis, disease diagnosis and treatment, energy, wastewater treatment, remediation, and antibacterial activity in recent years. Herein, iron-based nanoparticles (FeNPs), metallic nanoparticles, were synthesized via a facile chemical reduction method using a hyperaccumulator plant. Also, their use in antibacterial activity applications and colorimetric ascorbic acid (AA) detection was investigated. It was observed that FeNPs presented high antibacterial potency against Gram-positive bacteria of *Listeria monocytogenes* and *Staphylococcus aureus* and also Gram-negative bacteria of *Escherichia coli(O157: H7)*, *E. coli(ATCC 25922)*, *Salmonella enteritidis*, and *Salmonella typhimurium*. Moreover, it was found that FeNPs exhibited superior peroxidase-like activity to catalyze the oxidation of 3,3′,5,5′-tetramethylbenzidine (TMB) to produce a blue color product, oxidized TMB (oxTMB), in the presence of H_2_O_2_. The colorimetric AA detection could be carried out by making the solution color lighter owing to the antioxidant property of AA. The quantitative detection of AA could be performed simply, selectively, and sensitively with FeNPs with a detection limit (LOD) of 0.5462 μM in a linear range of 30–200 μM.

## 1 Introduction

Nanoparticles can be synthesized by physical, chemical, and biological methods or hybrid methods, which are combinations of several of them. In this study, a new nanoparticle synthesis method was developed that combines the advantages of chemical and biological methods. According to this method, the iron-based nanoparticle (FeNP) synthesis can be carried out by the chemical reduction of the metal ions in the leach solution prepared from the iron hyperaccumulator plant of *Pinus brutia*. With this developed method, it can be possible to synthesize nanoparticles in a short time with high efficiency, which is one of the advantages of chemical methods, and also the process cost and the formation of toxic byproducts can be reduced using plants, which is one of the advantages of biological methods. In the literature, the metallic nanoparticles synthesized by various methods could be effectively used in various application areas such as bioremediation and decontamination applications,^1^ biomedical applications,^2^ drug delivery,^3^ removal of various pollutants,^4^ catalyst applications,^5^ sensing applications,^6^ antibacterial activity applications,^7^ battery applications.^8^ Among the metallic nanoparticles, iron-based nanoparticles have attracted much attention due to their outstanding properties such as high reactivity, adsorption capacity, biocompatibility, and mechanical, chemical, and thermal stability.^9^ Additionally, they possess magnetic features, providing easy separation from the environment via an external magnet.^10^ Because of the as-mentioned superior properties of iron-based nanoparticles, they have been widely utilized in the applications of catalysis, antimicrobial, anticancer, biosensor, energy, and wastewater treatment.^11^ To the best of our knowledge, it is the first time to evaluate iron-based nanoparticles, synthesized using a hyperaccumulator plant as a metal ion source, in antibacterial activity applications and colorimetric AA detection in this study.

The researchers have focused on developing more effective antibacterial nanoparticles without side effects that are easy to enforce since the risk of biological and bacterial assaults, especially in the sectors of food, food packaging, and water, has increased gradually in recent years. The dimensions of metallic nanoparticles are between bulk materials and molecules/atoms/ions, which interact with cells to make stable entities with less energy.^12^ So, one of the most featured practical applications of iron-containing nanoparticles, metallic nanoparticles, is their utilization as antibacterial agents in the literature.^13−15^ In this study, the antibacterial activity of FeNPs synthesized from a hyperaccumulator plant was investigated against various food-borne bacteria.

Ascorbic acid (AA) is a natural water-soluble vitamin, which is a puissant reducing and antioxidant agent that has important roles in battling bacterial infections, detoxifying reactions, and the formation of collagen in fibrous tissue, teeth, bones, connective tissue, skin, and capillaries. AA concentrations in the human bloodstream are generally between 0.6 and 2.0 mg/dL; however, it varies from tissue to tissue. The deficiency of ascorbic acid (<0.4 mg/dL) in the body can cause the emergence of various diseases such as immunity decrease, anemia, and even scurvy. Taking more than the recommended amount of AA, which is more than 2.0 g per day, can result in side effects such as nausea and vomiting, diarrhea, abdominal pain and cramps, heartburn, insomnia, headache, fatigue, and kidney stones. Besides, AA has been gradually utilized in industries due to its strong reducing and antioxidant capacity.^16^ Therefore, it is crucial to detect AA with a simple, convenient, inexpensive, and sensitive method. Various methods have been established and applied to detect AA, including titration, enzymatic methods, electrochemical techniques, fluorescence and chemiluminescence methods, capillary electrophoresis, and chromatography methods.^17,18^ The detection of AA has been able to apply these methods with good sensitivities; however, these methods have some disadvantages such as the need for trained technicians, the requirement of expensive equipment or chemicals, and impractical and time-consuming operations. Among the traditional methods, the colorimetric method based on a chromogenic substrate producing color upon oxidation in the presence of the natural enzymes is more ideal and effective in detecting AA. However, natural enzymes are too sensitive to extreme experimental conditions, and so they can lose their catalytic activity easily at strong acidic/basic pHs and high-temperature values. In addition, there are some drawbacks of natural enzymes such as high cost, low stability, and difficulty in storage.^17,19^ Nanozymes are inorganic nanomaterials with a more effective enzyme-like catalytic activity in comparison to natural enzymes because of their high catalytic activity, low cost, high stability, and wide range of applications.^20^ Hence, to overcome these drawbacks, various nanozymes such as palygorskite@Co_3_O_4_ nanocomposites,^21^ polyacrylonitrile–CuO nanoflowers,^22^ platinum nanoclusters,^23^ gold nanoparticles,^24^ cobalt-doped carbon quantum dots,^25^ Cu–Ag bimetallic nanoparticles,^26^ and Fe_3_O_4_/nitrogen-doped carbon hybrid nanofibers^27^ have been developed as peroxidase-like catalysts to catalyze the oxidation of TMB in the presence of H_2_O_2_ for the colorimetric AA detection in the past decade. Since some difficulties have been faced with the sensitive detection of AA in complex biological media, the researchers are still focusing on improving the catalytic activity, sensitivity, selectivity, and stability of enzyme-mimetic nanomaterials. In this study, FeNPs synthesized from a hyperaccumulator plant were used instead of a natural enzyme in the oxidation reaction of TMB in the presence of H_2_O_2_, and the addition of the antioxidant agent AA to this reaction media enables simple, sensitive, and selective colorimetric detection of AA.

## 2 Materials and Methods

### 2.1 Synthesis and Characterization of Iron-Based Nanoparticles

According to the iron-based nanoparticle (FeNP) synthesis method developed by our team, NaBH_4_, as a reductant agent, was added to the leach solution prepared from *P. brutia* instead of the synthetic iron salt solution under the required experimental conditions. The details of the synthesis method and the results of some characterization studies were presented in our previous work.^28^ In this study, additional characterization studies using a Zeta-sizer via the dynamic light scattering technique (DLS), Fourier transform infrared (FTIR) spectra, and a vibrating sample magnetometer (VSM) were used to define the synthesized FeNPs.

### 2.2 Antibacterial Activity Test

*Salmonella enteritidis*, *Listeria monocytogenes*, *Escherichia coli* O157:H7, *E. coli* (ATCC 25922), *Staphylococcus aureus*, *andSalmonella typhimurium* bacteria species, which are widely used and food-borne pathogens, were selected to determine the antibacterial activities of FeNPs. In the scope of the culture and inoculum preparation, the bacterial cultures were grown on tryptic soy agar (TSA) slants and kept at 4 °C. Isolated colonies obtained from the TSA slants were inoculated into a tryptic soy broth (TSB) medium. The broth culture was incubated at 37 °C for 24 h. The optical density of the culture was adjusted between 0.08 to 0.1 at 625 nm to obtain an inoculum size of 1 × 10^7^ colony-forming unit (CFU)/mL. The antibacterial activity of FeNPs was investigated using the agar plate method. The surface of the agar plate (Mueller Hinton Agar) was inoculated by spreading the test microorganism over the entire surface. Then, a hole with a diameter of 6 mm was punched aseptically, and 0.0015 g of the test compound (FeNPs) and 30 μL of sterile distilled water were introduced into the well. The Petri dishes were incubated at 37 °C for 24 h. The test compound diffused into the agar and inhibited the growth of the test microorganism. The diameters of inhibition growth zones were measured with a digital caliper.^29^

### 2.3 Colorimetric Detection of Ascorbic Acid with FeNPs

The antioxidant property of AA was utilized for the colorimetric detection of AA. For this purpose, 500 μL of an acetate buffer solution (pH = 2.0), 500 μL of a 0.1 mM H_2_O_2_ solution, and 100 μL of a 1.0 g/L FeNP solution were added sequentially onto 250 μL of a 0.5 mM 3,3′,5,5′-tetramethylbenzidine (TMB) solution in an UV–vis spectrophotometer cuvette. This solution was named “control” by us. After that, the spectrum scanning of the “control” containing the oxidation products formed as a result of the oxidation of TMB was performed with a UV–vis spectrophotometer at a 300–1100 nm wavelength range. Then, a series of control solutions were prepared, and 1000 μL of AA solutions at different concentrations (1.0–500 μM) were added separately to each prepared control solution. The reductions in the absorption peak intensities were determined with the addition of AA by performing repeat spectrum scans of the prepared solutions in the wavelength range of 300–1100 nm. For the determination of the minimum limit of detection (LOD) of FeNPs, the absorbance values of the control solutions containing the oxidation products (*A*_i_) and AA solutions at different concentrations were added (*A*_f_) were recorded with a UV–vis spectrophotometer at a 652 nm wavelength. In order to find the LOD value, a calibration line was formed by plotting the different concentrations of AA against the absorbance changes (Δ*A* = *A*_i_—*A*_f_) at these concentrations. The limit of detection (LOD = 3σ/s) was calculated according to the signal, which is equivalent to 3 times the standard deviation of the blanks, where s is the slope of the calibration line and σ is the standard deviation of the control solution (the solution containing the oxidation products without adding AA).^21,30^

### 2.4 Determination of Selectivity of FeNPs

The colorimetric detection of AA with FeNPs was carried out in the presence of different components that can be found in real samples such as NaCl, KCl, CuCl_2_, CaCl_2_, ZnCl_2_, MgCl_2_, Al(NO_3_)_3_, (NH_4_)_2_HPO_4_, glucose, lactose, maltose, fructose, sucrose, urea, uric acid, ascorbic acid, oxalic acid, lactic acid, l-cysteine, glutathione, dopamine, and melamine. To determine the selectivity of FeNPs, 500 μL of an acetate buffer solution (pH = 2.0), 500 μL of a 0.1 mM H_2_O_2_ solution, and 100 μL of a 1.0 g/L FeNP solution were added sequentially into 250 μL of a 0.5 mM TMB solution, the solution containing all of the components was defined as “control”, in a UV–vis spectrophotometer cuvette. The absorbance values of the control solutions containing the oxidation products were recorded with a UV–vis spectrophotometer at a 652 nm wavelength. After that, the solutions of NaCl, KCl, CuCl_2_, CaCl_2_, ZnCl_2_, MgCl_2_, Al(NO_3_)_3_, (NH_4_)_2_HPO_4_, glucose, lactose, maltose, fructose, sucrose, urea, uric acid, ascorbic acid, oxalic acid, lactic acid, l-cysteine, glutathione, dopamine, and melamine were added to each prepared control solution as a mixture with AA (500 μL of 1.0 mM AA + 500 μL of 1.0 mM interferents). The absorbance values of the final solutions containing different interferents were recorded with a UV–vis spectrophotometer at a 652 nm wavelength, and the absorbance changes (Δ*A* = *A*_i_*– A*_f_) were calculated to determine the selectivity of FeNPs and the interactions of different components.^21,30^

The recovery values were calculated with eq 1 given below
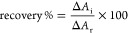
1where *A*_i_ is the absorbance change in the presence of the interferent i and *A*_r_ is the absorbance change in the presence of reference that is distilled water.

### 2.5 Method Validation

In order to test the applicability of the colorimetric detection of AA with FeNPs in real samples, commercial AA-containing materials (vitamin C tablets and vitamin C water) and AA solutions with known theoretical concentrations were used. For this purpose, the “control solution” was prepared, and the absorbance values of the control solution containing the oxidation products were recorded with a UV–vis spectrophotometer at a 652 nm wavelength. After that, a series of control solutions were prepared, and 1000 μL of the solutions (solution containing a vitamin C tablet, vitamin C water, and AA solutions with known theoretical concentrations) was added to the control solutions containing the oxidation products. The absorbance values of the final solutions containing AA were recorded with a UV–vis spectrophotometer at a 652 nm wavelength, and the absorbance changes (Δ*A* = *A*_i_*– A*_f_) were calculated. The concentration values of AA were calculated from the obtained calibration line equation. Accordingly, the accuracy analysis of the method was performed by comparing the theoretical and calculated concentration values.^21,30^ The formulae for the recovery value and the relative standard deviation (RSD) used in this analysis are given in eqs 2 and 3, respectively
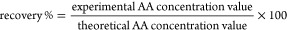
2
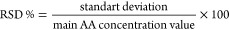
3

## 3 Results and Discussion

### 3.1 Characterization of FeNPs

The characterization results of XRD, EDX, and SEM of the synthesized FeNPs were presented in our previous work.^28^ The XRD analysis results showed that FeNPs contained the crystal phases of Fe_3_O_4_, FeOOH, γ-FeOOH, and Fe^0^. The average particle size of FeNPs was obtained as 82.19 nm, and also the nearly spherical structures were observed in SEM images. It was determined from EDX analysis that FeNPs contained the elements of O, Fe, Ca, Na, Mg, and Cl in descending order.

**Figure 1:**
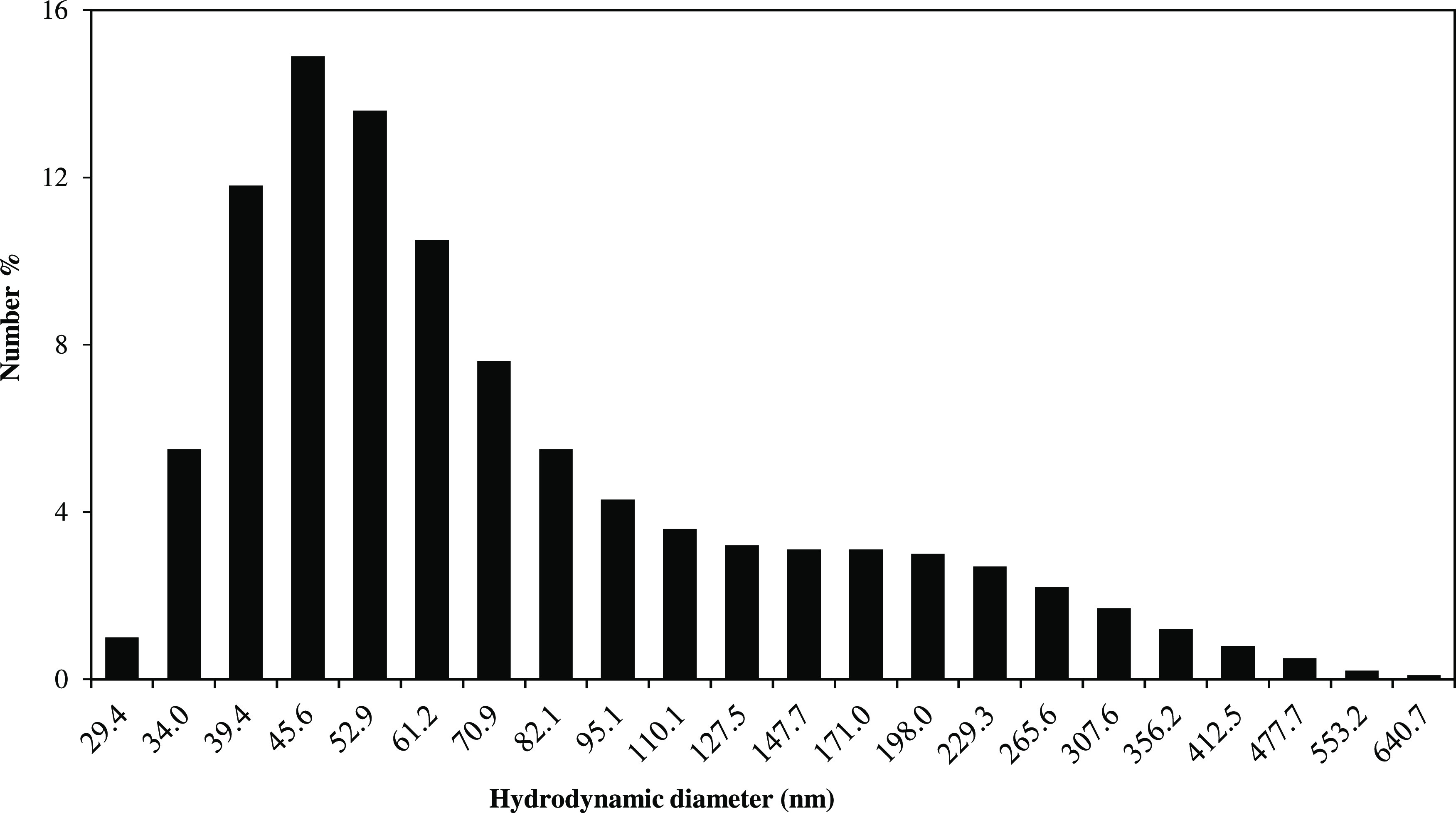
Particle size distribution of FeNPs (FeNP synthesis conditions: 5.0 mL of a leach solution (13.81 mg/L Fe ion), pH ≈ 1.18, 65 °C, 0.175 g NaBH_4_).

In this study, additional characterization studies such as FTIR, DLS, and VSM were carried out to determine the functional groups, the hydrodynamic diameter, and the magnetic properties of FeNPs. The hydrodynamic diameter of FeNPs was measured with a zeta-sizer via the DLS technique, and the average hydrodynamic diameter was found to be approximately 94 nm. The particle size distribution is also demonstrated in Figure 1. From Figure 1, it was observed that the particles with the sizes of 100–200 nm were obtained in the DLS analysis as a result of the agglomeration of the small particles. On the other hand, the structures larger than 200 nm are estimated to be the particles containing Ca, Na, Mg, and Cl elements originating from the hyperaccumulator plant used in the synthesis of FeNPs, which were also detected in the EDX analysis in our previous work.^28^

**Figure 2:**
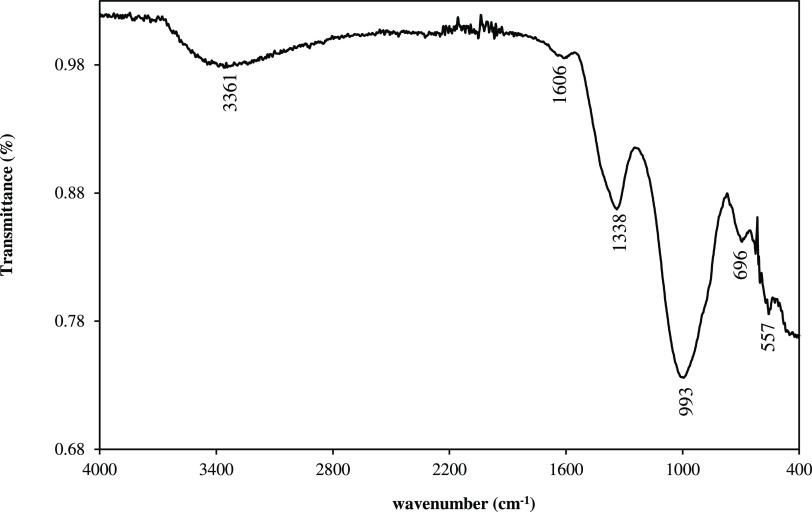
FTIR spectrum of FeNPs (FeNP synthesis conditions: 5.0 mL of the leach solution (13.81 mg/L Fe ion), pH ≈ 1.18, 65 °C, 0.175 g NaBH_4_).

The functional groups of the synthesized FeNPs were determined by FTIR analysis, and the obtained FTIR spectrum is presented in Figure 2. It was obtained from Figure 2 that FeNPs had peaks at 557, 696, 993, 1338, 1606, and 3361 cm^–1^. The broad absorption bands at 3361 and 1606 cm^–1^ were assigned to the stretching and bending vibrations of hydroxyl groups and/or water molecules, respectively. FeNPs had peaks at 557 and 1338 cm^–1^, referring to the vibration and stretching of the Fe–O bond. The −OH bending caused by Fe–OH groups appeared at 696 and 993 cm^–1^.^31−34^

**Figure 3:**
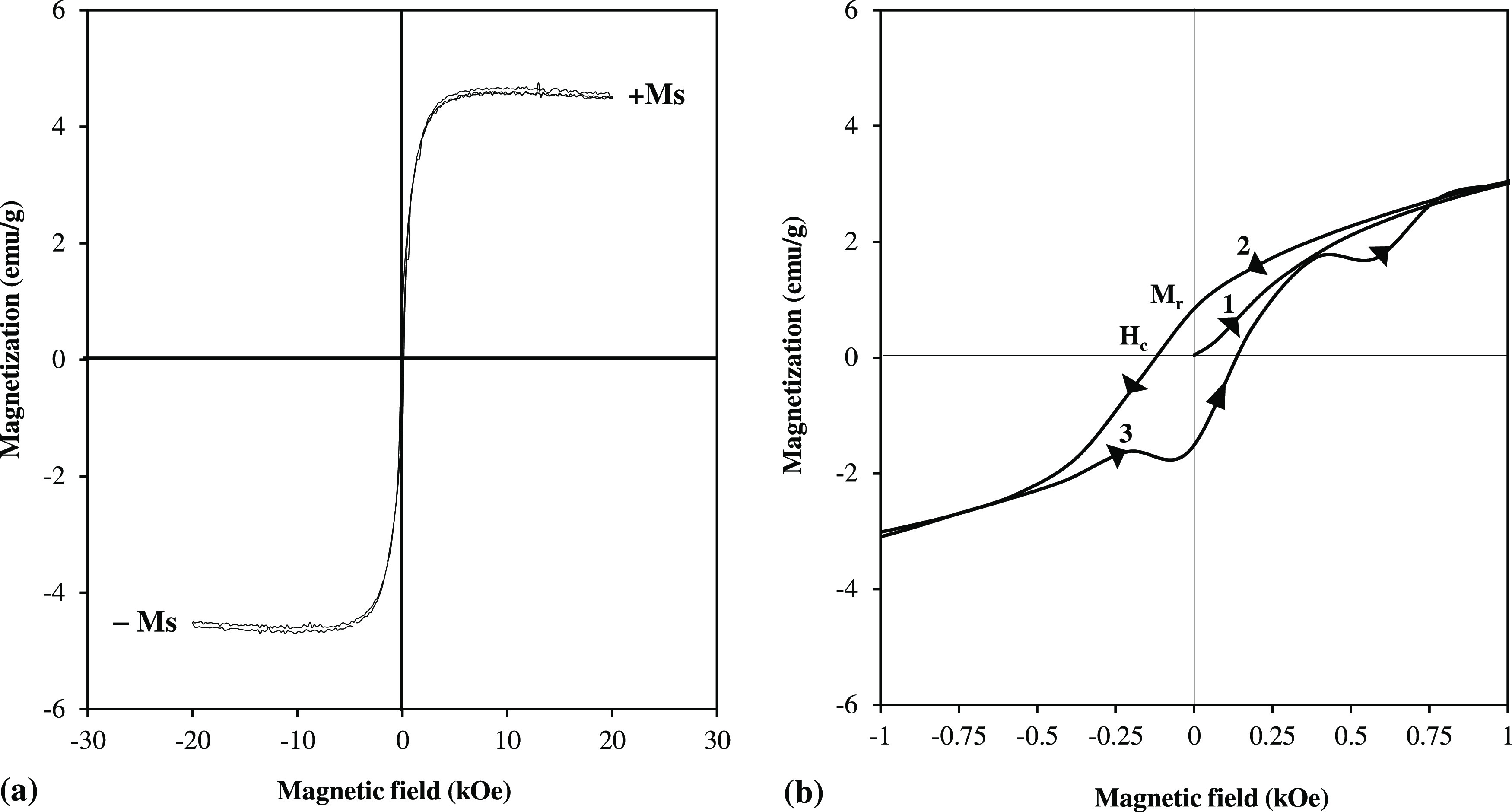
VSM analysis of FeNPs (a) in the magnetic field range of ± 20 kOe and (b) in the magnetic field range of ± 1.0 kOe (FeNP synthesis conditions: 5.0 mL of the leach solution (13.81 mg/L Fe ion), pH ≈ 1.18, 65 °C, 0.175 g NaBH_4_).

A vibrating sample magnetometer (VSM) was used to determine the magnetic properties of FeNPs by measuring the magnetization versus the applied magnetic field (M–H) curve at room temperature in the magnetic field range of ± 20 kOe. The hysteresis loops shown in Figure 3a,b indicated that FeNPs were ferromagnetic in nature. The saturation magnetization (*M*_s_), remanent magnetization (*M*_r_), and coercivity (*H*_c_) values were obtained to be 4.5 emu/g, 0.8438 emu/g, and 125 Oe, respectively. The remanent magnetization value (*M*_r_ < 1.125) obtained for FeNPs, less than 25% of the *M*_s_ value, showed that FeNPs could be easily separated from the media with a permanent magnet and could be quickly dissolved in the solution without agglomeration. Since the *H*_c_ value of FeNPs synthesized in this study was 125 Oe, it was determined that FeNPs were classified as semihard magnetic materials. It was concluded that FeNPs, which were classified as semihard magnetic materials, required more energy than soft materials to move in the loop and required a lower magnetic field than hard magnetic materials to reach saturation magnetization. Semihard magnetic materials have a wide range of uses such as magnetically coupled devices (brakes, clutches, tensioners), bias elements in product protection/safety systems, relay magnets, magnetic tool holders, sensor magnets, magnetic stirrers, and level sensors.^35−37^ Accordingly, it can be said that FeNPs synthesized in this study could be used in the application of semihard magnetic materials.

The saturation magnetization value (*M*_s_) of FeNPs was compared with the iron-containing nanoparticles in the literature, and the results are summarized in Table 1. According to Table 1, it was determined that FeNPs synthesized in this study had a lower *M*_s_ value than iron-containing nanoparticles in the literature. It was thought that other components except for iron in FeNPs obtained in EDX analysis reduced the saturation magnetization (*M*_s_) value of FeNPs.

**Table 1:** Saturation Magnetization Values (*M*_s_) of Iron-Containing Nanoparticles in the Literature

ion-containing nanoparticles	*M*_s_(emu/g)	refs
Fe_3_O_4_ nanoparticles (commercial)	92	(38)
citric acid-functionalized iron oxide nanoparticles	90.23	(39)
ferromagnetic Fe_3_O_4_	73.1	(40)
green synthesized Fe_3_O_4_ nanoparticles	73.04	(38)
Fe_3_O_4_ nanoparticles	65.53	(41)
Fe_3_O_4_ nanoparticles coated with pentaerythritol tetrakis(3-mercaptopropionate)-polymethacrylic acid	45	(42)
FeOOH/γ-Fe_2_O_3_ nanoparticles	36.4	(43)
Fe_3_O_4_ nanoparticles	20.639	(44)
iron oxide-hydroxyapatite nanocomposite	7.34	
green synthesized Fe_3_O_4_ nanoparticles	17.3	(45)
poly-methylmethacrylate 10% Fe nanoparticles	11.5	(46)
poly-methylmethacrylate 1% Fe nanoparticles	0.411	
green synthesized iron oxide nanoparticles	5.35	(47)
green synthesized Fe_3_O_4_ nanoparticles	5.14	(48)
FeNPs	4.5	this work
Zn-doped α-Fe_2_O_3_ nanoparticles	3.81	(49)
reductive-co-precipitated cellulose immobilized zerovalent iron nanoparticles	3.0	(50)
gluconic acid-capped iron oxide nanoparticles	2.69	(51)
magnetic iron nanoparticles	1.5	(52)
biosynthesized iron oxide nanoparticles	0.3414	(53)

### 3.2 Antibacterial Activity of FeNPs

**Figure 4:**
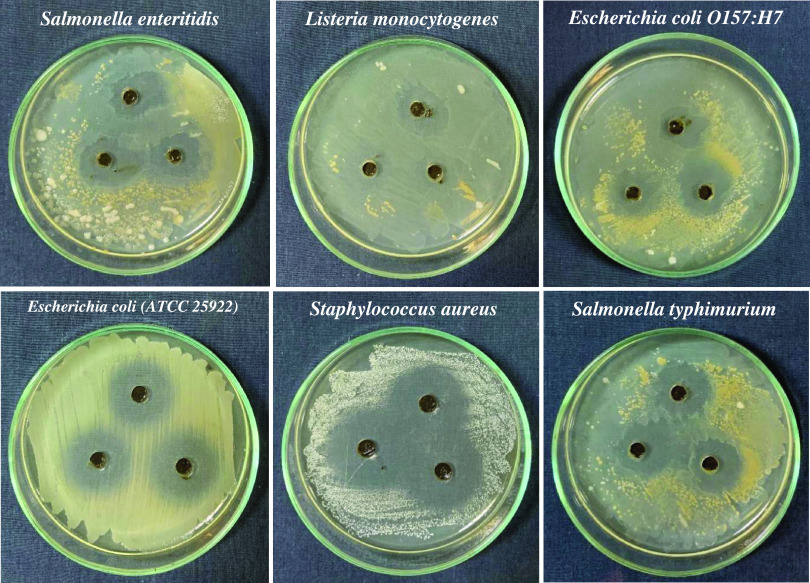
Images showing the antibacterial activity of FeNPs in test microorganisms.

**Figure 5:**
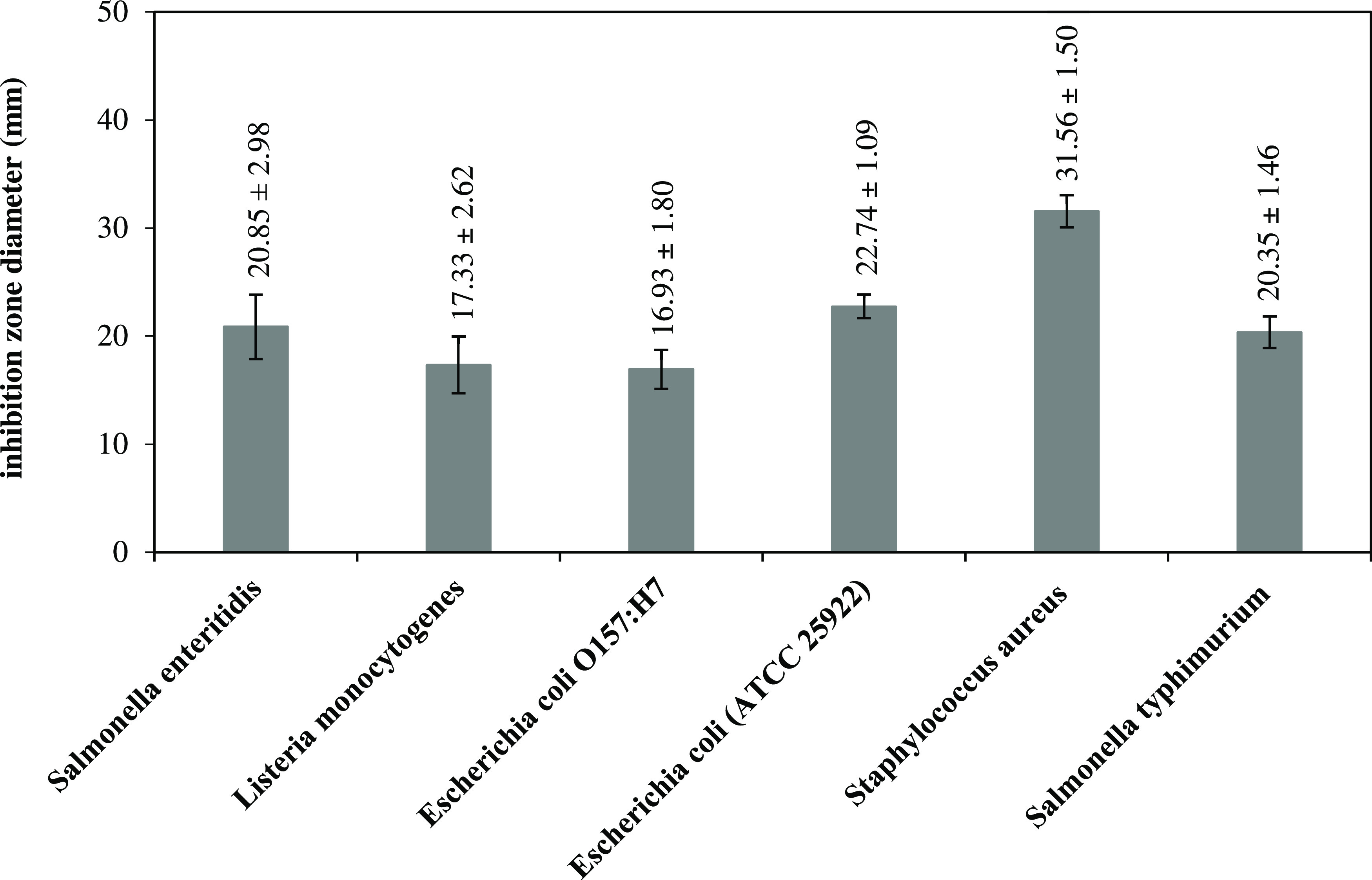
Graph showing the inhibition zone diameters obtained for each microorganism.

The images showing the antibacterial activity of FeNPs on test microorganisms and the graph showing the inhibition zone diameters obtained for each microorganism are presented in Figures 4 and 5, respectively. The inhibition zone diameters of the sample compound for the bacterial species of *S. enteritidis*, *L. monocytogenes*, *E. coliO157:H7*, *E. coli(ATCC 25922)*, *S. aureus*, and *S. typhimurium* were determined as 20.85 ± 2.98, 17.33 ± 2.62, 16.93 ± 1.80, 22.74 ± 1.09, 31.56 ± 1.50, and 20.35 ± 1.46 mm, respectively. Accordingly, it was proved that FeNPs were effective against all selected bacteria. As a result, it was concluded that the strong inhibitory effect of FeNPs synthesized in this study against the tested food-borne microorganisms will allow FeNPs to be used as a preservative agent in the food industry to reduce food contamination and provide longer-lasting storage. In this case, it can be recommended to use FeNPs synthesized from the hyperaccumulator plant of *P. brutia* for bacterial-resistant coating and antibacterial applications for biomedical devices.

The inhibition zone diameters of FeNPs were compared with the iron-containing nanoparticles in the literature, and the results are summarized in Table 2. It was concluded from Table 2 that the FeNPs synthesized in this study were at a competitive level with various iron-containing nanoparticles in the literature in terms of antibacterial activity.

**Table 2:** Comparison of Inhibition Zone Diameters of FeNPs with the Iron-Containing Nanoparticles in the Literature

nanoparticles	synthesis method	microorganism	inhibition zone diameter (mm)	refs
iron oxide nanoparticles (20 μg/mL)	sol–gel	*E. coli*	22	(39)
		*Bacillus subtillis*	27	
iron oxide nanoparticles (30 μg/mL)	biosynthesis with the plant extract	*B. subtilis*	10 ± 0.20	(54)
		*E. coli*	18 ± 0.34	
		*Klebsiella pneumoniae*	16 ± 0.40	
		*S. aureus*	13 ± 0.23	
Fe_3_O_4_(100 μg/mL)	chemical combustion	*S. aureus*	15	(55)
		*Xanthomonas*	15	
		*E. coli*	21	
		*Proteus vulgaris*	21	
iron oxide nanoparticles (20 mg/mL)	biosynthesis with plant extract	*S. aureus*	8	(56)
		*E. coli*	10	
iron oxide nanoparticles (50 mg/mL)	coprecipitation	*S. aureus*	12 ± 0.35	(57)
		*Bacillus licheniformis*	22 ± 0.70	
		*Bacillus brevis*	9 ± 0.15	
		*Vibrio cholerae*	9 ± 0.0	
		*Streptococcus aureus*	12 ± 0.35	
		*Staphylococcus epidermidis*	14 ± 0.44	
		*B. subtilis*	20 ± 1.11	
		*E. coli*	11 ± 0.44	
FeNPs (50 mg/mL)	reduction method developed by us	*S. enteritidis*	20.85 ± 2.98	this work
		*L. monocytogenes*	17.33 ± 2.62	
		*E. coliO157:H7*	16.93 ± 1.80	
		*E. coli* (*ATCC 25922*)	22.74 ± 1.09	
		*S. aureus*	31.56 ± 1.50	
		*S. typhimurium*	20.35 ± 1.46	
iron nanoparticles	biosynthesis with the plant extract	*E. coli*	27	(58)
		*Pseudomonas aeruginosa*	29	
		*S. aureus*	30	
iron nanoparticles	biosynthesis with the plant extract	*E. coli*	1.60 ± 0.40	(59)
		*S. aureus*	1.90 ± 0.10	
		*P. aeruginosa*	1.00 ± 0.40	
		*B. subtillis*	5.05 ± 0.05	
iron nanoparticles	biosynthesis with the fungal biomass	*B. subtillis*	16.4 ± 0.70	(60)
		*S. aureus*	12.3 ± 0.50	
		*E. coli*	13.2 ± 0.60	
		*P. aeruginosa*	10.5 ± 0.30	
iron nanoparticles	biosynthesis with the plant extract	*E. coli*	15	(61)
		*Salmonella enterica*	12	
		*Proteus mirabilis*	13	
		*S. aureus*	16	

### 3.3 Colorimetric Detection of Ascorbic Acid with FeNPs

**Figure 6:**
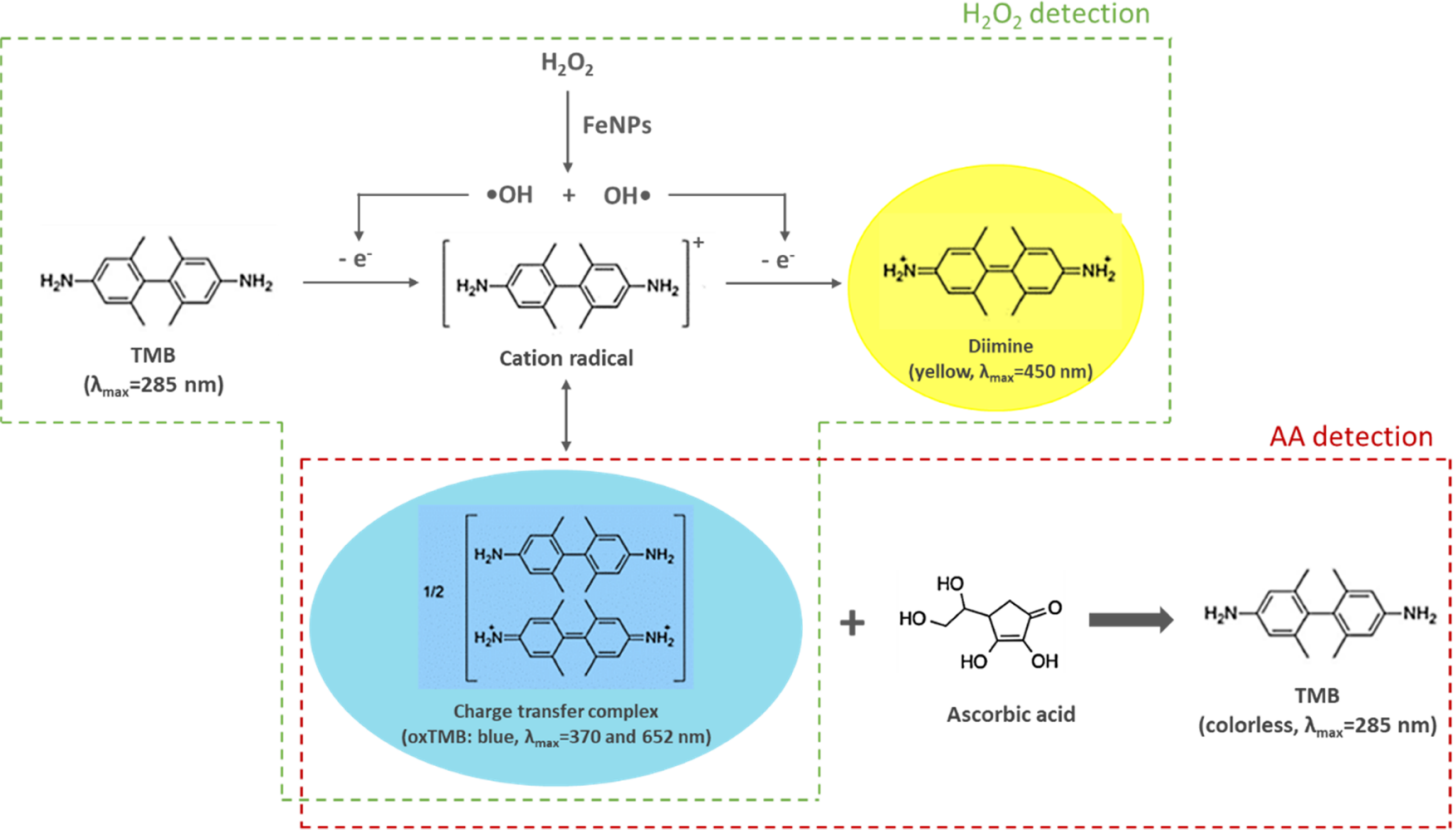
Predicted colorimetric AA detection steps.

**Figure 7:**
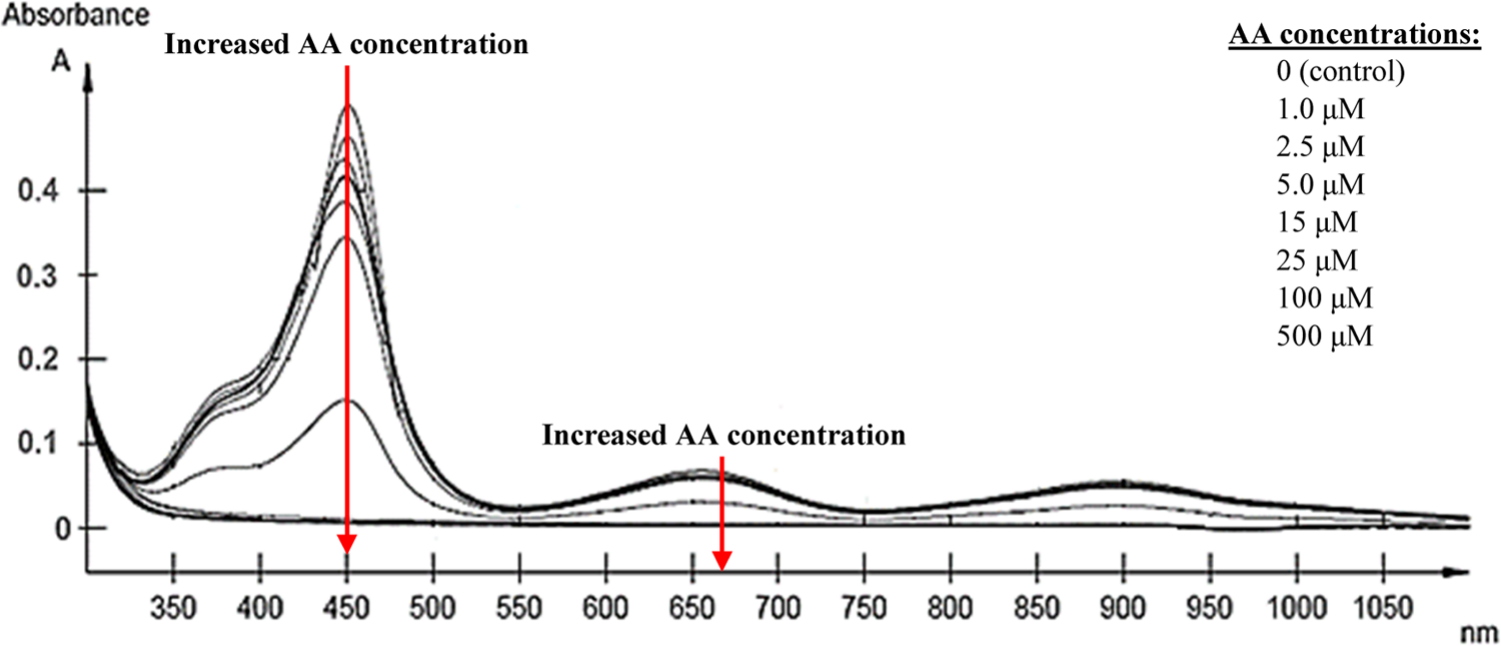
UV–vis spectra with the change in the AA concentrations (1.0–500 μM).

According to the predicted colorimetric AA detection steps given in Figure 6, FeNPs could catalyze the decomposition of H_2_O_2_ via a Fenton-like reaction to generate ^•^OH radicals, which could oxidize the chromogenic substrate TMB to the oxidized TMB (oxTMB). The colorimetric detection of H_2_O_2_ with FeNPs as a peroxidase-like catalyst could be done by the spectrophotometric analysis of the colored oxidation products formed at the end of this reaction. For the detection of AA with FeNPs, AA could discolor the blue color of oxTMB to colorless-TMB by means of the antioxidant property of AA. The UV spectrum scans of the solutions given in Section 2.3 containing the oxidation products of TMB were performed to verify the colorimetric AA detection steps. As shown in Figure 7, the absorbance intensities at 450 and 652 nm decreased gradually with the increase in AA concentrations from 1.0 to 500 μM, along with the solution color changing from blue to colorless. The color changes in 5 s with increasing AA concentration could also be visualized through the naked eye, discoloring from blue to colorless.

**Figure 8:**
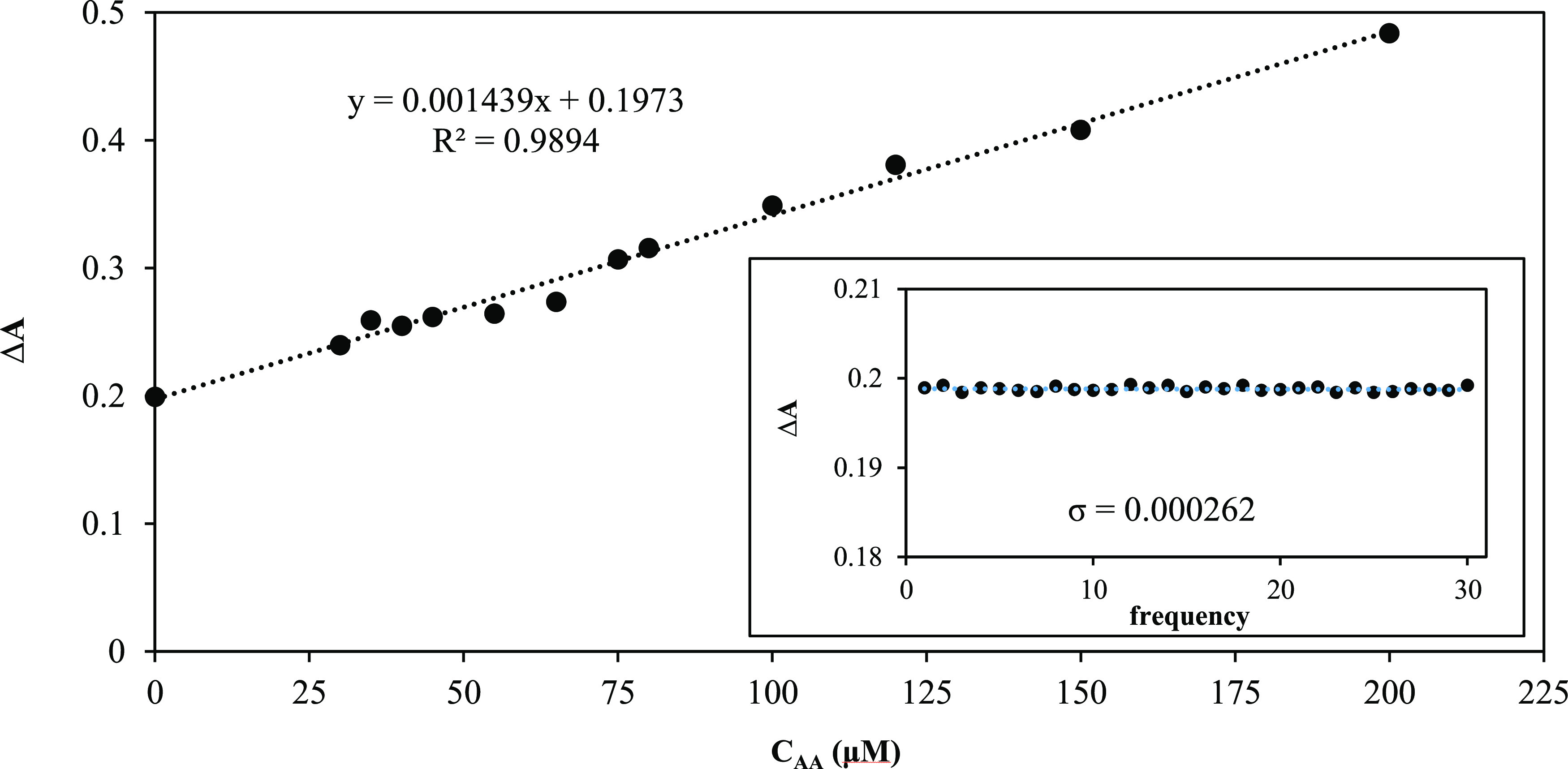
Calibration line for the colorimetric detection of AA with FeNPs (inset: the obtained absorbance changes for the blanks).

In order to determine the LOD value of FeNPs for the colorimetric detection of AA via TMB oxidation in the presence of H_2_O_2_, a calibration line given in Figure 8 (inset, the plot used to calculate the standard deviation value of the control solution) was formed by plotting the AA concentrations against the absorbance changes. The absorbance changes at 652 nm had a good linear regression equation Δ*A* = 0.001439 × *C*_AA_ (μM) + 0.1973 (*R*^2^ = 0.9894) with the AA concentration in a range of 30–200 μM. The limit of detection (LOD) of FeNPs for the colorimetric AA detection was calculated to be 0.5462 μM at an S/N (signal/noise) ratio of 3.0.

Table 3 summarizes the comparison of FeNPs synthesized in this work with currently available peroxidase-like nanomaterials for colorimetric AA detection. As shown in Table 3, both the linear range and LOD of the synthesized FeNPs are comparable to or even better than some of the reported peroxidase-like nanomaterials synthesized from synthetic metal ion sources in the literature. These results demonstrated that the proposed FeNPs could be evaluated as effectively as peroxidase-like nanomaterials synthesized from synthetic metal ion sources in the literature.

**Table 3:** Comparison of the Linear Range and the Minimum Detection Limit of Different Nanomaterials in the Literature

nanomaterials	linear range (μM)	LOD (μM)	refs
CoOOH nanomaterials	0.01–1.0	0.005	(62)
Fe_3_O_4_/nitrogen-doped carbon hybrid nanofibers	0–50	0.04	(27)
MnO_2_ nanosheets	0.25–30	0.063	(30)
Pt/CeO_2_ nanocomposites	0.5–30	0.08	(63)
cobalt oxyhydroxide nanoflakes	0.5–50	0.142	(64)
reduced graphene oxide nanosheets functionalized with poly(styrene sulfonate)	0.8–70	0.15	(65)
Co_3_O_4_ nanoparticles/crumpled graphene microsphere	30–140	0.19	(66)
carbon dots/Fe_3_O_4_ hybrid nanofibers	1.0–30	0.285	(67)
FeNPs	30–200	0.5462	this work
polyacrylonitrile–copper oxide nanoflowers	1.0–180	0.56	(22)
palygorskite@Co_3_O_4_ nanocomposites	1.0–60	0.70	(21)
CuO–Pt nanocomposites	1.0–600	0.796	(68)
3,4:9,10-perylene tetracarboxylic acid modified litchi-like zinc ferrite nanocomposites	1.0–10	0.834	(69)
Fe–Mn bimetallic nanozymes	8.0–56	0.88	(17)
hollow mesoporous carbon nanospheres loaded with Pt nanoparticles	6.0–60	3.29	(70)
Cu–Ag bimetallic nanoparticles on reduced graphene oxide nanosheets	0.005–0.03	3.60	(26)

### 3.4 Determination of Selectivity of FeNPs

**Figure 9:**
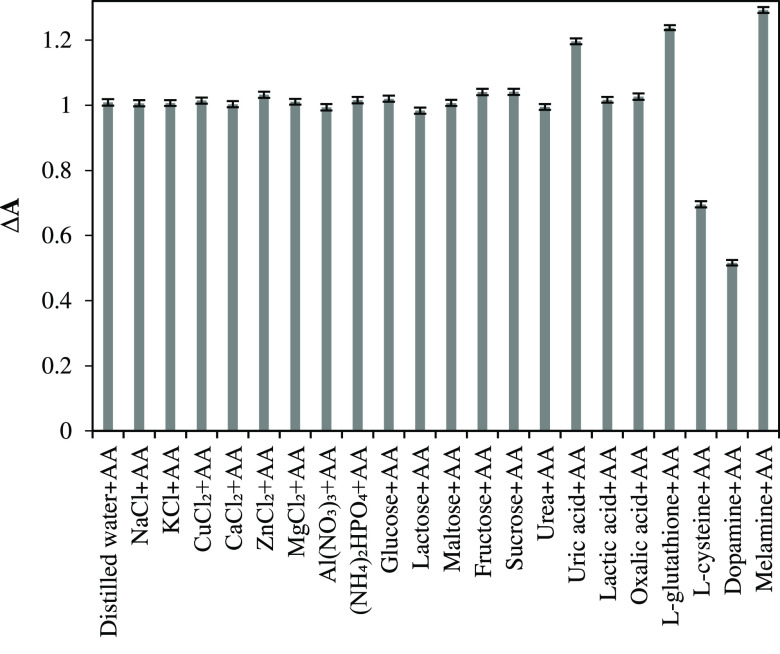
Selectivity of FeNPs for the colorimetric AA detection via TMB oxidation in the presence H_2_O_2_.

The possible interfering substances such as NaCl, KCl, CuCl_2_, CaCl_2_, ZnCl_2_, MgCl_2_, Al(NO_3_)_3_, (NH_4_)_2_HPO_4_, glucose, lactose, maltose, fructose, sucrose, urea, uric acid, ascorbic acid, oxalic acid, lactic acid, l-cysteine, l-glutathione, dopamine, and melamine were selected for the determination of selectivity of FeNPs. It is obvious from Figure 9 that the absorbance changes in the presence of NaCl, KCl, CuCl_2_, CaCl_2_, ZnCl_2_, MgCl_2_, Al(NO_3_)_3_, (NH_4_)_2_HPO_4_, glucose, lactose, maltose, fructose, sucrose, urea, ascorbic acid, oxalic acid, and lactic acid were approximately same with the control experiment using the distilled water. Furthermore, as can be seen in Table 4, the recovery values for the substances of NaCl, KCl, CuCl_2_, CaCl_2_, ZnCl_2_, MgCl_2_, Al(NO_3_)_3_, (NH_4_)_2_HPO_4_, glucose, lactose, maltose, fructose, sucrose, urea, ascorbic acid, oxalic acid, and lactic acid were between 97.46 and 103.17%. These results revealed that the colorimetric AA detection could be carried out successfully with FeNPs synthesized from the hyperaccumulator plant of *P. brutia* in the presence of various interfering substances except for uric acid, l-cysteine, l-glutathione, dopamine, and melamine.

**Table 4:** Recovery Values of the Possible Interfering Substances

substances	recovery %
NaCl + AA	99.68
KCl + AA	99.73
CuCl_2_ + AA	100.54
CaCl_2_ + AA	99.41
ZnCl_2_ + AA	102.27
MgCl_2_ + AA	100.16
Al(NO_3_)_3_ + AA	98.50
(NH_4_)_2_HPO_4_ + AA	100.64
glucose + AA	101.06
lactose + AA	97.46
maltose + AA	99.79
fructose + AA	103.10
sucrose + AA	103.17
urea + AA	98.59
uric acid + AA	117.88
lactic acid + AA	100.75
oxalic acid + AA	101.72
l-glutathione + AA	122.69
l-cysteine + AA	69.17
dopamine + AA	51.29
melamine + AA	128.84

### 3.5 Method Validation

For the investigation of the reliability of the colorimetric AA detection with FeNPs, the method was applied to analyze AA content in real samples. As presented in Table 5, the recovery values were from 100.59 to 104.57%, and RSD % values were in the range of 1.14–3.28%. The results confirmed that the colorimetric AA detection method with FeNPs synthesized from the hyperaccumulator plant of *P. brutia* possessed the potential to be applied accurately and reliably to the detection of AA content in real samples.

**Table 5:** Method Validation Results

sample	theoretical concentration (μM)	experimental concentration (μM) (*n* = 3)	recovery %	RSD % (*n* = 3)
AA solution	30	31.37 ± 1.03	104.57	3.28
vitamin C water (commercial)	45.42	45.92 ± 1.36	101.10	2.96
AA solution	60	60.95 ± 1.13	101.58	1.85
AA solution	120	120.71 ± 1.87	100.59	1.55
solution containing a vitamin C tablet (commercial)	142	143.56 ± 1.63	101.10	1.14

## 4 Conclusions

In summary, we first developed a new facile method using the leach solution of hyperaccumulator plant *Pinus brutia* as a natural metal ion source to synthesize novel iron-based nanoparticles for the antibacterial activity and the colorimetric detection of ascorbic acid. The results showed that FeNPs exhibited a significant bactericidal effect toward Gram-positive (*L. monocytogenes* and *S. aureus*) and Gram-negative (*E. coli(O157: H7)*, *E. coli(ATCC 25922)*, *S. enteritidis*, and *S. typhimurium*) pathogenic bacteria. The findings suggested that the leach solution prepared from the iron hyperaccumulator plant of *Pinus brutia* could be used for developing antibacterial FeNPs against pathogenic bacteria. Furthermore, a sensitive and selective colorimetric AA detection system was successfully constructed using FeNPs as enzyme mimics. This colorimetric detection system using FeNPs could be applied to quantify AA concentration, with a linearity range of 30–200 μM and an LOD value of 0.5462 μM. It was observed that the colorimetric AA detection could be effectively carried out in the presence of various interfering substances except for uric acid, l-cysteine, l-glutathione, dopamine, and melamine. It was also tested to quantify AA in real samples, and their recovery values were 100.59–104.57% with RSD less than 4%, proving its feasibility in evaluating AA content in practical application. Considering all of these, the present work not only provides a novel antibacterial agent and a peroxidase-like catalyst but also inspires researchers to further explore various hyperaccumulator plants for the synthesis of metallic nanoparticles for a variety of applications.
